# In this month’s Bulletin

**DOI:** 10.2471/BLT.24.000824

**Published:** 2024-08-01

In the editorial section this month, Yohei Sasakawa (554) explains what’s needed to achieve zero leprosy. Kristina Jenei and Veronika J Wirtz (555) argue for continued measurement of access to essential medicines in the sustainable development goals.­

Gary Humphreys (558–559) reports on a decade of water and sanitation efforts in India. Carlos Monteiro talks to Gary Humphreys (560–561) about Brazil’s dietary transition and the need for substantial change at every level of food production, marketing and consumption to address obesity.

## Bangladesh 

### How to count unplanned pregnancies

Md Nuruzzaman Khan et al. (562–570) compare two survey methods for creating estimates.

## Chile, Finland, Italy, Malta, Mexico, Peru, United Kingdom of Great Britain and Northern Ireland, United States of America

### National health examination surveys

Paula Margozzini et al. (588–599) advocate for this critical source of data.

## Croatia, Finland, France, Germany, Greece, Ireland, Italy, Latvia, Lithuania, Luxembourg, Portugal, Slovakia, Spain, United Kingdom of Great Britain and Northern Ireland 

### Pandemic preparedness and health system resilience 

Kaitlyn Hall Radford et al. (571–581) review national plans.

## Kenya

### Reducing consumption of cigarettes, vapes and oral nicotine pouches 

Cyprian M Mostert et al. (618–620) put forward arguments for tax increases.

## Global

### Non-partner sexual violence against women

Lynnmarie Sardinha et al. (582–587) critique global prevalence estimates.

### Improving tuberculosis treatment

Christian Lienhardt et al. (600–607) list characteristics of new target regimens profiles.

### Sporting events and pandemic risks 

Albis Francesco Gabrielli et al. (608–614) describe risk-based management approaches used during the COVID-19 pandemic.

### Genetic variants causing *glucose-6-phosphate dehydrogenase* deficiency

Lucio Luzzatto et al. (615–617) detail changes to WHO’s classification.

